# Therapeutic strategies for spinal muscular atrophy: SMN and beyond

**DOI:** 10.1242/dmm.030148

**Published:** 2017-08-01

**Authors:** Melissa Bowerman, Catherina G. Becker, Rafael J. Yáñez-Muñoz, Ke Ning, Matthew J. A. Wood, Thomas H. Gillingwater, Kevin Talbot, The UK SMA Research Consortium

**Affiliations:** 1 Department of Physiology, Anatomy and Genetics, University of Oxford, South Parks Road, Oxford OX1 3QX, UK; 2 Euan MacDonald Centre for Motor Neurone Disease Research and Centre for Neuroregeneration, University of Edinburgh, Edinburgh EH16 4SB, UK; 3 AGCTlab.org, Centre for Biomedical Sciences, School of Biological Sciences, Royal Holloway, University of London, Egham Hill, Egham, Surrey TW20 0EX, UK; 4 Department of Neuroscience, Sheffield Institute for Translational Neuroscience (SITraN), University of Sheffield, Sheffield S10 2HQ, UK; 5 Euan MacDonald Centre for Motor Neurone Disease Research and Centre for Integrative Physiology, University of Edinburgh, Edinburgh EH8 9XD, UK; 6 Nuffield Department of Clinical Neurosciences, University of Oxford, John Radcliffe Hospital, Oxford OX3 9DU, UK

**Keywords:** Animal models, Cellular models, Combinatorial therapies, Skeletal muscle, Spinal muscular atrophy, Survival motor neuron

## Abstract

Spinal muscular atrophy (SMA) is a devastating neuromuscular disorder characterized by loss of motor neurons and muscle atrophy, generally presenting in childhood. SMA is caused by low levels of the survival motor neuron protein (SMN) due to inactivating mutations in the encoding gene *SMN1*. A second duplicated gene, *SMN2*, produces very little but sufficient functional protein for survival. Therapeutic strategies to increase SMN are in clinical trials, and the first *SMN2*-directed antisense oligonucleotide (ASO) therapy has recently been licensed. However, several factors suggest that complementary strategies may be needed for the long-term maintenance of neuromuscular and other functions in SMA patients. Pre-clinical SMA models demonstrate that the requirement for SMN protein is highest when the structural connections of the neuromuscular system are being established, from late fetal life throughout infancy. Augmenting SMN may not address the slow neurodegenerative process underlying progressive functional decline beyond childhood in less severe types of SMA. Furthermore, individuals receiving SMN-based treatments may be vulnerable to delayed symptoms if rescue of the neuromuscular system is incomplete. Finally, a large number of older patients living with SMA do not fulfill the present criteria for inclusion in gene therapy and ASO clinical trials, and may not benefit from SMN-inducing treatments. Therefore, a comprehensive whole-lifespan approach to SMA therapy is required that includes both SMN-dependent and SMN-independent strategies that treat the CNS and periphery. Here, we review the range of non-SMN pathways implicated in SMA pathophysiology and discuss how various model systems can serve as valuable tools for SMA drug discovery.

## Abstract (precis)

**Summary:** Translational research for spinal muscular atrophy (SMA) should address the development of non-CNS and survival motor neuron (SMN)-independent therapeutic approaches to complement and enhance the benefits of CNS-directed and SMN-dependent therapies.

## Introduction

**Fig. 1.**
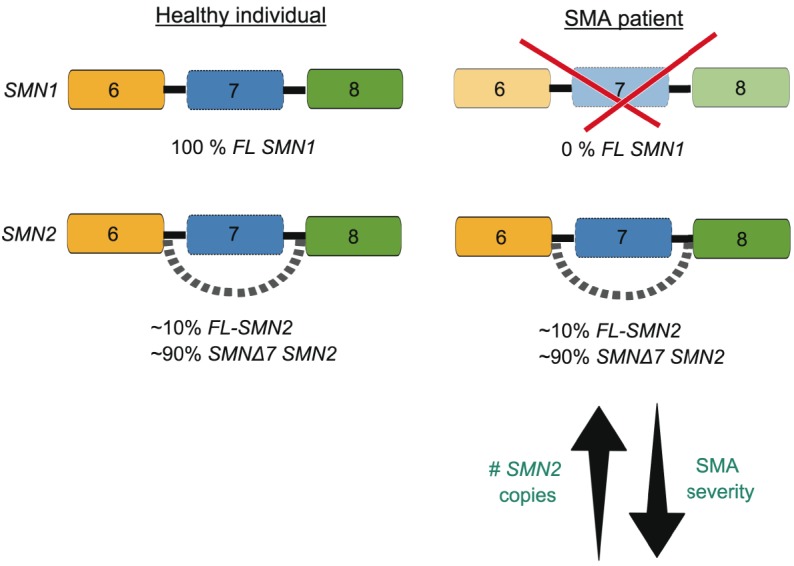
***SMN1* and *SMN2* contribute to spinal muscular atrophy (SMA).** In healthy individuals, the survival motor neuron 1 (*SMN1)* gene produces 100% full length (FL) SMN protein while the *SMN2* gene produces ∼10% FL SMN and ∼90% of a non-functional product that lacks exon 7 (SMNΔ7) due to aberrant alternative splicing. In SMA patients, the *SMN1* gene is lost due to mutations or deletions. *SMN2* remains and the small amount of FL SMN is sufficient for survival. The number of *SMN2* copies correlates with disease severity, with a lower copy number being linked to more severe types of SMA.

Spinal muscular atrophy (SMA) is the most common genetic disease resulting in death in infancy, affecting approximately 1 in 6000 to 1 in 10,000 births (Crawford and Pardo, 1996). This autosomal recessive disorder, resulting from the loss-of-function of the survival motor neuron 1 (*SMN1*) gene, is characterized by loss of spinal cord motor neurons, muscular atrophy, neuromuscular junction (NMJ) denervation and paralysis (Crawford and Pardo, 1996; Kariya et al., 2008; Kong et al., 2009; Lefebvre et al., 1995; Murray et al., 2008). *SMN1* is highly conserved and present as a single copy in the genome of all eukaryotic organisms (Bergin et al., 1997; Miguel-Aliaga et al., 1999, 2000; Paushkin et al., 2000). In humans, however, a genomic duplication has given rise to a second gene, *SMN2* (Lefebvre et al., 1995; Rochette et al., 2001). A crucial C-to-T substitution at position 6 of exon 7 in *SMN2*, which occurs in all individuals, leads to the aberrant splicing of exon 7 and the subsequent production of an unstable SMNΔ7 protein (Lorson et al., 1999; Monani et al., 2000). An important sequence in intron 7 of *SMN2*, termed intron splicing silence N1 (ISS-N1) has been demonstrated to further favour the exclusion of exon 7 in the transcript (Singh et al., 2006). Thus, the telomeric *SMN1* copy gives rise to the full-length (FL) SMN protein while the centromeric *SMN2* copy predominantly produces the SMNΔ7 protein. However, the *SMN2* gene always generates a small amount of functional protein, which maintains viability, as homozygous deletion of *SMN1* is uniformly lethal (Gennarelli et al., 1995; Lefebvre et al., 1995). Deletions or intragenic mutations in *SMN1* are found in all forms of SMA, with *SMN2* acting to modulate the disease severity through variation in its copy number (Gennarelli et al., 1995; Lefebvre et al., 1995) (Fig. 1). As the number of copies of *SMN2* increases, so does the quantity of stable FL-SMN protein produced. Thus, the variation in clinical severity seen in SMA is mostly explained by the total level of residual SMN protein.

SMA is clinically heterogeneous and has been categorized into five types (0-IV) based on age of onset, severity of motor decline and life expectancy. The term ‘Type 0’, in which the minimal complement of one *SMN2* gene is present, describes SMA with a clear *in utero* onset, arthrygryphosis (limited joint contracture) and complex motor and sensory nerve deficits, and death before or just after birth. Type I SMA patients display the most severe symptoms, with death in infancy if invasive ventilation is not implemented. Types II and III have a later childhood onset and are associated with survival into adulthood, and the potential for a normal lifespan, albeit with considerable physical disability (Munsat and Davies, 1992; Pearn, 1980). The clinical course of Type II and III patients living with SMA is characterized by long periods of relative stability with superimposed periods of accelerated functional decline, for example during the pubertal growth spurt, and a subsequent long period of slowly progressive age-related loss of motor function (Kaufmann et al., 2012). Advances in respiratory, musculoskeletal and nutritional care mean that greater numbers of patients with Type I SMA are surviving beyond infancy. Most SMA Type II patients are living full lives into adulthood and Type III SMA is, in the majority of cases, associated with a normal lifespan (Rudnik-Schöneborn et al., 2001). The clinical and molecular features of the five types of SMA are presented in Table 1. Given the range of severity and ages of onset, it will be necessary for any therapeutic strategy to address the needs of all individuals affected by SMA, from infancy to adulthood.

**Table 1.**
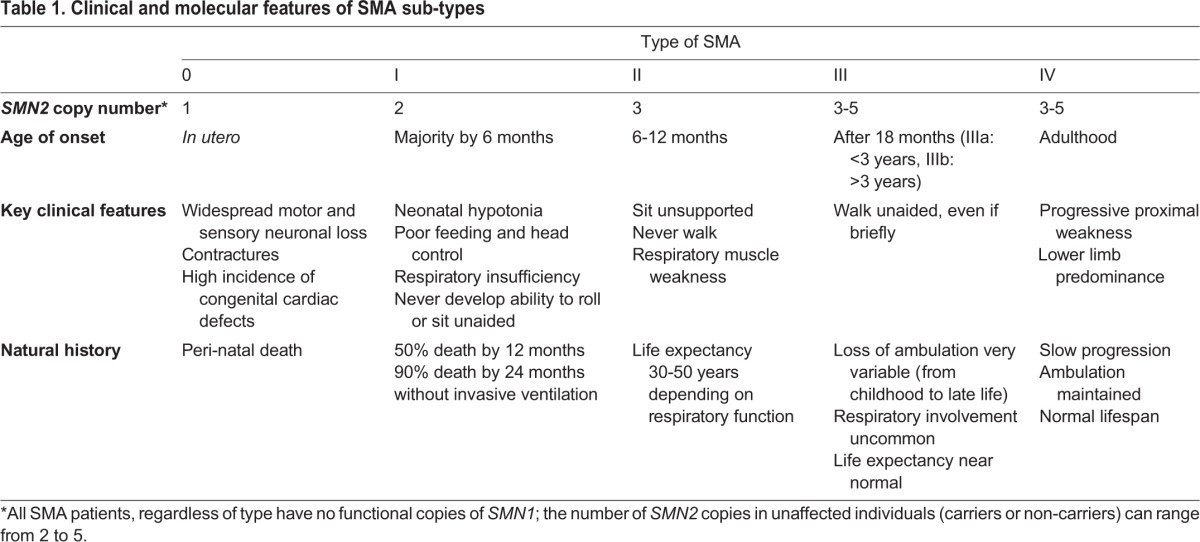
**Clinical and molecular features of SMA sub-types**

**Fig. 2.**
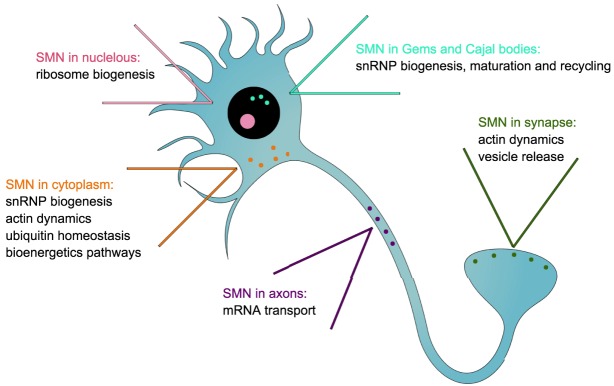
**Localization of the survival motor neuron (SMN) protein in neuronal cells and associated general cellular functions.** SMN regulates small nuclear ribonucleoprotein (snRNP) biogenesis, maturation and recycling in Gemini of coiled bodies (Gems) and Cajal bodies; ribosome biogenesis in nucleolus; snRNP biogenesis and actin dynamics in the cytoplasm; mRNA transport in axons; actin dynamics and vesicle release in the synpase.

The SMN protein is ubiquitously expressed and is localized in the cytoplasm (Liu and Dreyfuss, 1996), neuronal growth cones (Fan and Simard, 2002), neuronal extensions (Fallini et al., 2010), the nucleolus (Charroux et al., 2000; Wehner et al., 2002) and in punctate nuclear structures called Gemini of coiled bodies (Gems) and Cajal bodies (Carvalho et al., 1999; Liu and Dreyfuss, 1996). The SMN protein has thus been attributed several key regulatory cellular functions in neuronal cells, including roles in RNA metabolism [specifically small nuclear ribonucleoproteins (snRNPs)] (Li et al., 2014), actin cytoskeleton dynamics (Hensel and Claus, 2017), mRNA transport (Donlin-Asp et al., 2016), ubiquitin homeostasis (Groen and Gillingwater, 2015), bioenergetics pathways (Boyd et al., 2017) and synaptic vesicle release (Kong et al., 2009) (Fig. 2). Importantly, to date, none of these roles has been identified as being solely responsible for SMA pathophysiology.

The most advanced therapies currently in clinical trials for SMA are aimed at increasing FL SMN either by exogenously expressing *SMN1* or upregulating FL *SMN2* production (d'Ydewalle and Sumner, 2015). Unless these current SMN-dependent approaches can be given pre-symptomatically, when motor neuron dysfunction may still be reversible, and delivered with a very high level of efficiency to drastically induce SMN levels in spinal cord motor neurons, it is likely that the progressive neurodegenerative process will not be completely abrogated but simply slowed down. Thus, treated SMA patients may be vulnerable to a delayed deterioration of the neuromuscular system. There are also a large number of older children and adults living with SMA who do not fulfill the present criteria for inclusion (on the grounds of age and various clinical parameters) in the ongoing clinical trials and for whom it is not currently clear that SMN-inducing treatments will be beneficial because of the significant and irreversible SMN-related neuromuscular decline that is already established. Furthermore, it has become evident that SMA pathophysiology extends beyond the neuromuscular system, whereby numerous peripheral organs and tissues demonstrate pathological changes in pre-clinical models and patients (Hamilton and Gillingwater, 2013). Therapies that improve neuromuscular function as well as maintain lifelong general health of people living with SMA are therefore a major priority and an unmet clinical need.

As the first successful SMN-targeted therapeutic approaches are emerging into the clinical arena (Finkel et al., 2016; Gillingwater, 2016), we review here how to best move forward in the development of combinatorial therapeutic approaches for SMA that, ideally, would target the CNS and the periphery, operating via SMN-dependent and SMN-independent processes. We will firstly consider the various existing and alternative experimental models that could be used to identify novel SMA therapeutic targets. We will then discuss how the current SMN-specific compounds presently in clinical trials inform the potential development of treatments aimed at non-SMN targets in non-CNS tissues. Finally, we will expand on the idea of developing drug discovery and delivery approaches that enable systemic delivery of therapies.

## Advantages and limitations of current animal and cell models

### Animal models

A range of *in vivo* model systems have been developed to aid understanding of the pathogenesis of SMA and to test the efficacy of therapies. The similarities in anatomy and physiology to the human neuromuscular system, coupled with the ease of genetic manipulation, mean that the mouse has been an extremely valuable model for exploring the basic pathogenesis and evaluating potential treatments for SMA. Various mouse models have been developed over the years, displaying differing ranges of disease severity. While the complete knockout (*Smn^−/−^*) is embryonic lethal (Schrank et al., 1997), the heterozygous animals (*Smn^+/−^*) do not develop a typical SMA phenotype (Bowerman et al., 2014; Schrank et al., 1997), which is in accordance with data demonstrating that loss of ∼85% of normal Smn levels is required to reflect an SMA phenotype in mice (Bowerman et al., 2012a). Genetic modifications were thus integrated in the *Smn^–/–^* mice to allow their survival, whilst still retaining the criteria for an SMA model. The severely afflicted *Smn^–/–^;SMN2* mice (Hsieh-Li et al., 2000; Monani et al., 2000) harbor a human *SMN2* transgene that produces the typical ∼15% FL *SMN2* transcript, whereas the *Smn^–/–^;SMN2;SMN^Δ7/Δ7^* transgenic mice (Le et al., 2005) additionally express the partially functional human SMNΔ7 protein, which confers an increase in survival. Both these severe SMA mouse models typically do not survive past the first two post-natal weeks and therefore do not reflect the chronic phase of the disease, making them suitable for modelling only severe infantile SMA. This limitation means that these mice are also unsuitable for the evaluation of non-SMN therapies that may benefit some aspects of SMA pathology. In addition, severe SMA mouse models do not allow for the long-term evaluation of extra-CNS defects that might emerge over time in people living with Type II, III and IV SMA. To address this, animals that have a significantly longer asymptomatic phase have been developed. *Smn^2B/–^* mice (Bowerman et al., 2012a; Hammond et al., 2010) are an intermediate model with an average lifespan of 30 days. These mice carry an endogenous mutation within the murine *Smn* gene that mimics the human *SMN2* gene by principally producing *SMNΔ7* transcripts. Recently, longer-lived intermediate models were generated by administration of sub-optimal doses of exon 7 inclusion-promoting ISS-N1 antisense oligonucleotides (ASOs, Box 1) to human *SMN2*-carrying SMA mice (to promote an insufficient increase of FL-SMN for complete rescue) (Hosseinibarkooie et al., 2016; Zhou et al., 2015). Mouse models thus remain an important system for augmenting our understanding of the SMA disease process and in the evaluation of potential therapeutic approaches.

**Box 1. Glossary**

**AAV:** adeno-associated virus. A small DNA virus that infects humans and other primate species without causing disease.

**ASO:** antisense oligonucleotide. A synthetic polymer, typically 15-20 nucleotides long and complementary to the sense sequence of a target mRNA.

**BBB:** blood-brain barrier. A highly selective semi-permeable endothelial cell barrier separating the circulating blood from the brain in the central nervous system.

**CPP:** cell-penetrating peptide. A short peptide that facilitates cellular uptake of molecules.

**Discordant:** in disagreement.

**FDA:** Food and Drug Administration of the United States Department of Health and Human Services; responsible for protecting and promoting public health.

**Hypomorphic:** genetic mutation causing partial loss of function.

**Intracerebroventricular:** direct injection into the ventricular system (i.e. into the cerebral ventricles) of the brain.

**Intrathecal:** direct injection into the sub-arachnoid space so that a drug reaches the cerebrospinal fluid.

**NMJ:** neuromuscular junction. The chemical synapse between a motor neuron and a muscle fibre.

**SMN:** survival motor neuron protein encoded by the human *SMN1* and *SMN2* genes that are ubiquitously expressed in most cells and tissues with a range of cell-specific and housekeeping functions.

More targeted hypotheses about the role of SMN and its interaction with specific proteins can be successfully explored in invertebrates, particularly *Drosophila* (fruit flies) (Chan et al., 2003; Chang et al., 2008) and *Caenorhabditis elegans* (Burt et al., 2006), and genetically tractable vertebrate model organisms that present a developmental phenotype, such as zebrafish (Hao et al., 2011; McWhorter et al., 2003). However, these models may display phenotypes that differ greatly from what is observed in mouse models and SMA patients. The locomotor and motility defects characterized in *Drosophila* larval SMA models (Praveen et al., 2012, 2014) can only have an indirect relationship to the disruptions in neuromuscular function that occur in patients. In zebrafish models of SMA generated by antisense morpholinos or maternal zygotic genetic mutations (Hao et al., 2013; McWhorter et al., 2003), developing motor axons and dendrites display outgrowth and branching defects, whereas in mouse and human, SMA motor axons correctly reach the target muscle and form the NMJ, followed by denervation of the muscle as the disease progresses (Ling et al., 2012). Nevertheless, such model systems are more efficient than mammalian models for high-throughput screening and are likely to have significant advantages if utilized efficiently and thoughtfully.

Finally, there have also been initiatives to develop large animal models for SMA, particularly for the evaluation of the delivery, benefits and toxicity of clinical-grade therapeutics. Specifically, endogenous and exogenous genetic modifications have been introduced in the pig to generate a porcine SMA model (Duque et al., 2015; Towne et al., 2010). More work is needed to evaluate whether the pig will become the pre-clinical model of choice for therapeutic assessment.

### Cellular models

There are obvious limitations in the extent to which mouse models and the other *in vivo* models described above might be predictive of effects in humans, owing to inherent species-specific differences in cellular function. The inability to directly study neurons in individuals affected by a neurological disorder such as SMA has been a key impediment to understanding basic pathological mechanisms, particularly those occurring early in the disease process. Recent developments in stem cell technology have substantially expanded the range of cellular models available in motor neuron disease research by allowing the direct observation of pathological mechanisms in neurons derived from induced pluripotent stem cells (iPSCs) obtained from fibroblasts derived from affected individuals. SMA was the first neurological disorder in which a disease-relevant phenotype was demonstrated in iPSC-derived motor neurons (Ebert et al., 2009).

Beyond neurons, other cell types have been demonstrated to contribute to SMA pathology, as described below. iPSCs can be converted into pancreatic and cardiac cells, and thus represent an even more powerful tool, as these cell lines can be studied separately or in co-culture systems that mimic the dynamic interaction that exists in the human body (De Vos et al., 2016). Furthermore, iPSCs can also be used to generate endothelial cells (Lippmann et al., 2014) to model the blood-brain barrier (BBB; Box 1) that protects the CNS, but also limits the entry of therapeutic compounds (Abbott, 2013; Pardridge, 2005), thus allowing screening of novel and established drugs for the ability to cross the BBB as well as compare the properties of SMA and healthy endothelial cells.

**Fig. 3.**
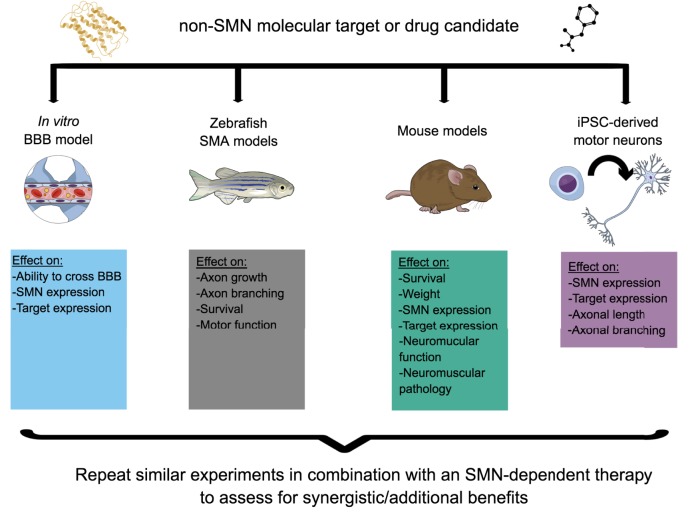
**A model experimental plan to determine the therapeutic potential of a candidate non-survival motor neuron (SMN) molecular target or drug, alone and as a combinatorial therapy.** Various *in vitro* and *in vivo* models such as an *in vitro* blood-brain barrier (BBB) model, *Smn* mutant zebrafish, hypomorphic SMA mouse models and induced pluripotent stem cell (iPSC)-dervied cells (e.g. motor neurons) could be used to evaluate different activity and efficiency parameters of the candidate target or drug. Each model system has a particular informative value depending on the candidate target or drug. Finally, the same experimental paradigm should be followed to assess the synergistic or additive value of combining the non-SMN treatment strategy with an SMN-dependent therapy.

iPSC-derived motor neurons can be generated from families in which genotypically matched individuals are discordant for the SMA phenotype, to serve as a tool to identify disease modifiers (Boza-Morán et al., 2015). Similarly, iPSCs can be generated from Type I, II and III patients, allowing the exploration of the impact of small changes in SMN expression at a cellular level. While iPSCs hold a lot of promise, experimental caveats include heterogeneity of the cell populations derived, as well as the limitations of studying cells in culture, isolated from other tissues within the context of the whole organism (Rouhani et al., 2014; De Filippis et al., 2017; Ebert et al., 2012). Nevertheless, iPSC-based models will give new insights into disease mechanisms and will also serve as a screening and validation tool for potential therapies identified in model organisms, especially as part of an experimental workflow designed to identify novel molecular targets and drugs and evaluate their combinatorial potential. Indeed, testing of candidate therapies across multiple platforms will most likely be key to the efficient and successful advancement of complementary therapeutic approaches (Fig. 3).

## Current SMN-targeted trials: early successes

At the forefront of SMA translational research are efforts now entering clinical trials for therapies that promote *SMN2* exon 7 inclusion via ISS-N1 inhibition, that increase *FL SMN2* transcription (small molecules) or that directly replace *SMN1* (viral gene therapy) (Table 2) (d'Ydewalle and Sumner, 2015). Two decades of basic research characterizing the molecular basis of exon 7 splicing (Singh et al., 2017) have recently culminated in the first successful clinical trial of an ASO therapy (nusinersen, commercial name Spinraza) developed and commercialized by Ionis Pharmaceuticals and Biogen (Finkel et al., 2016; Gillingwater, 2016). Not only did intrathecal delivery of nusinersen improve some disease symptoms in Type I patients, but there was also evidence that levels of FL SMN were increased in spinal motor neurons of treated individuals. Although this was an open-label (unblinded) trial, treatment can be cautiously compared favourably to the devastating natural history of Type I SMA patients where the majority of children have died or become ventilator-dependent before the age of 12 months. Whilst the results of an ongoing Phase III study in a larger cohort of patients are awaited, the drug was approved in December 2016 by the United States Food and Drug Administration (US FDA) for all types of SMA based on the strength of the existing data. Likewise, nusinersen was recommended for European Union approval by the EMA in April 2017 and given marketing authorization in June 2017.

**Table 2.**
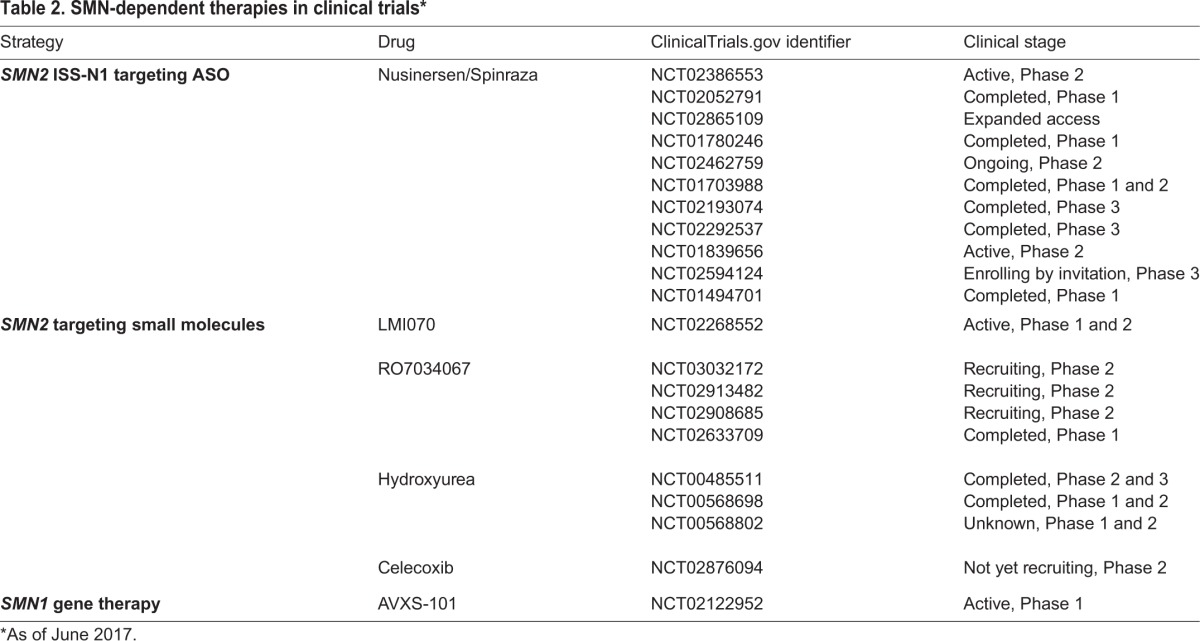
SMN-dependent therapies in clinical trials*

The ASO therapeutic approach, however, requires invasive intrathecal and intracerebroventricular administration for adequate delivery to the CNS, thus not addressing the issue of expression levels of FL SMN required in peripheral tissues, which are also potentially key contributors to SMA pathology (see below). A non-replicating adeno-associated virus (AAV9) vector has been developed by AveXis to deliver a functional copy of a human *SMN1* gene. A potential advantage of this approach is that AAV9 crosses the perinatal BBB, allowing a single intravenous dose to ensure widespread systemic delivery. A Phase 1 clinical trial is now underway. Small molecules that increase FL *SMN2* expression can also be administered systemically but their ability to cross the BBB may be limited and their exact mode of action needs to be deciphered to minimize potential off-target adverse effects. Small molecules targeting *SMN2* developed by Novartis Pharmaceuticals and Hoffmann-La Roche are currently in clinical trials.

Given that nusinersen and other small molecules act on *SMN2*, the evaluation of their activity in pre-clinical studies was limited to severe *SMN2-*harboring mice and patient fibroblasts (Pinard et al., 2017; Ratni et al., 2016; Singh et al., 2017). Similarly, the efficiency of AAV9-*SMN1* to increase SMN expression was demonstrated in mice with severe SMA (Armbruster et al., 2016; Passini et al., 2010). Whilst use of additional models such as *Drosophila* and zebrafish may not be relevant in these instances, assessing the activity of SMN-dependent approaches in iPSC-derived motor neurons, *in vitro* BBB models and other cell types may help shed light on cell-specific activity and efficiency of these various drugs, which could help in the optimization of their respective dosing regimens.

## Expanding the repertoire of targets to non-neuronal tissues

Although SMA has generally been considered as an archetypal disorder of selective motor neuron vulnerability, there is now abundant evidence that other tissues and cells are either overtly or sub-clinically affected in SMA patients and animal models (Hamilton and Gillingwater, 2013), including skeletal muscle (Boyer et al., 2013; Martínez-Hernández et al., 2009; Mutsaers et al., 2011; Walker et al., 2008), pancreas (Bowerman et al., 2012c, 2014), liver (Bowerman et al., 2012c, 2014; Szunyogova et al., 2016), spleen (Deguise et al., 2017; Thomson et al., 2017), vasculature (Somers et al., 2012, 2016), heart (Bevan et al., 2010; Heier et al., 2010; Shababi et al., 2010) and Schwann cells (Aghamaleky Sarvestany et al., 2014; Hao et al., 2015; Hunter et al., 2014, 2016). The clinical implications of these observations is that treatment modalities that solely target the nervous system might be an inadequate long-term therapeutic strategy for SMA, especially in Type I patients where the consequences of low levels of SMN for the function of non-CNS tissues beyond infancy is unknown.

To date, a single clinical trial provides evidence for the potential benefit of non-SMN therapies in the chronic phase of SMA. Olesoxime is an orally active drug, which is predicted to have favourable bioenergetics effects as it acts on the outer mitochondrial membrane to modulate the permeability transition pore opening in response to oxidative stress. It has a neuroprotective effect in a range of *in vitro* and *in vivo* models (Bordet et al., 2010). A recent clinical trial in SMA Type II and III (non-ambulatory) patients did not achieve its primary endpoint, although it showed some evidence of disease-stabilising effects using secondary endpoints (Bertini et al., 2017).

Pharmacological compounds aimed at specifically targeting skeletal muscle are presently the only non-SMN, non-CNS drugs in clinical trials for SMA patients. In particular, Cytokinetics Inc. has developed a fast skeletal troponin activator (CK-2127107) that is currently in a Phase 2 study in SMA patients. Troponin mediates muscle contraction (Gresslien and Agewall, 2016), and is abnormally distributed in skeletal muscle of Type I-III SMA patients (Stevens et al., 2008). Interestingly, CK-2127107 has not been evaluated in either pre-clinical or Phase 1 clinical stages as a therapeutic strategy for SMA. Instead, the drug was investigated in a rat model of heart failure, where its oral administration resulted in an overall amelioration of muscle endurance and performance following exercise (Hwee et al., 2015). A first generation form of the compound (tirasemtiv or CK-2017357) was also assessed in rodent neuromuscular models for nemaline myopathy (Lee et al., 2013), myasthenia gravis (Russell et al., 2012) and amyotrophic lateral sclerosis (Hwee et al., 2014), demonstrating a significant improvement in muscle strength in each. Nevertheless, the FDA has recently granted the Orphan Drug designation to CK-2127107 for the treatment of SMA patients.

Another interesting muscle target is the myostatin-follistatin pathway, in which myostatin acts as a negative regulator of muscle growth (McPherron et al., 1997) and is itself inhibited by follistatin (Lee and McPherron, 2001). Several therapeutics strategies aimed at modulating this signaling cascade to promote muscle mass have therefore been evaluated in pathologies characterized by muscle atrophy, including SMA (Rodino-Klapac et al., 2009). Administration of recombinant follistatin to *Smn^–/–^;SMN2;SMN^Δ7/Δ7^* mice resulted in significant improvement in muscle mass, gross motor function and lifespan. However, inhibition of myostatin by genetic (overexpression of follistatin) or pharmacological [soluble activin receptor IIB (ActRIIB-Fc) that inhibits the myostatin-promoting receptor] interventions had no obvious beneficial effects on the phenotype of the same mouse model (Sumner et al., 2009). Similar negative results were obtained upon genetically deleting myostatin in severe SMA mice (Rindt et al., 2012). Conversely, the recent intraperitoneal administration of a soluble form of ActRIIB encoded by an AAV2/8 viral vector in a milder model of SMA improved mass, contractile properties and size of SMN-depleted muscles (Liu et al., 2016). Results from SMA mouse studies have therefore been varied and the discrepancies may be due to the differing severities of the models used, the developmental timing of the approach and the specific targeting/delivery strategy utilized. Several strategies based on the myostatin-follistatin pathway are currently in clinical trials for muscle pathologies such as Duchenne muscular dystrophy (DMD), Becker muscular dystrophy and inclusion body myositis but have yet to be initiated for SMA. However, a recent report shows that serum and muscle biopsies from SMA patients display decreased expression of myostatin and increased levels of follistatin (Mariot et al., 2017), suggesting that additional mechanistic insight in the relevance of targeting this pathway for SMA therapy is required.

Taken together, these findings highlight an important need to better understand the intrinsic pathologies not only in SMA neurons but also in muscle and other non-CNS afflicted organs, so that cell- and tissue-specific treatments can be developed and eventually used in combination with SMN- and CNS-targeted strategies.

## Identifying non-SMN targets to develop combinatorial therapeutic approaches

SMN-dependent gene therapies will require administration as early as possible, even pre-symptomatically, to exert the maximum effect (Bevan et al., 2010; Foust et al., 2010; Valori et al., 2010), and at present can be expected to reduce disease severity rather than effect a complete cure. In the absence of a routine screening program for newborns, the potential benefit may also be limited by the delay in diagnosis in milder forms, which generally have an insidious onset. An important step forward would be to develop therapeutic approaches targeting pathways that reflect the chronic pathological process in SMA, facilitating treatments that are adjunctive to SMN replacement therapy to improve and maintain neuromuscular integrity and function throughout the life of the individual.

One of the first SMN-independent targets to prove beneficial with respect to potential SMA therapy is the RhoA-ROCK pathway. The small GTPase RhoA and its downstream effector, the Ser/Thr protein kinase ROCK, are key modulators of actin dynamics (Luo et al., 1997). It has been demonstrated that the RhoA-ROCK pathway is aberrantly upregulated in SMN-depleted rodent neuronal cells (Bowerman et al., 2007) and in the spinal cord and skeletal muscle of *Smn^2B/–^* mice (Bowerman et al., 2010, 2012b). Importantly, it was shown that pharmacological inhibition of ROCK significantly increases lifespan and muscle pathology of *Smn^2B/–^* SMA mice (Bowerman et al., 2010, 2012b). Additional investigators have further confirmed the contribution of the RhoA-ROCK pathway to SMA in neuronal cells (Hensel et al., 2014), patient fibroblasts (Nölle et al., 2011) and glial cells (Caraballo-Miralles et al., 2012). The tumour suppressor protein PTEN is a member of the protein tyrosine phosphatase family that can regulate cell migration, spreading and growth (Lachyankar et al., 2000; Li et al., 2003; Sano et al., 1999; Tamura et al., 1999). Interestingly, PTEN is phosphorylated by ROCK (Bermúdez Brito et al., 2015), thus leading to increased PTEN inhibitory activity on neuronal survival. While PTEN activity in SMA mice has yet to be investigated, we can hypothesize that the increased activity of the RhoA-ROCK pathway reported in SMA mice (Bowerman et al., 2010, 2012b) induces the increased phosphorylation of PTEN. Concordantly, it has been found that suppressing PTEN in SMA mice through a gene therapy approach led to improvements in NMJ pathology and a significant extension in lifespan (Little et al., 2015). Combined, these studies have highlighted actin modulators as potential targets for combinatorial therapeutic approaches for SMA.

Further regulators of actin dynamics have emerged as potential therapeutic targets for SMA. Plastin 3 (PLS3) is an actin-bundling protein that was identified as a modifier of disease severity in an investigation of discordant family members that carried the same *SMN1* mutations (Oprea et al., 2008). Additional analyses of serum (Yanyan et al., 2014) and iPSC-derived motor neurons from SMA patients have further supported the influence of plastin 3 levels on disease progression in certain families but not others (Boza-Morán et al., 2015; Heesen et al., 2016). Indeed, overexpression of plastin 3 in a zebrafish model of SMA significantly rescues the axonal growth and branching defects caused by *smn1* gene depletion (Oprea et al., 2008). Further analysis of *smn1* mutant zebrafish reveals that reduced SMN levels lead to decreased plastin 3 protein expression, NMJ defects and aberrant motor function, and these effects can be corrected by plastin 3 overexpression (Hao et al., 2012). More recently, studies in mice have shown that increased expression of plastin 3 delays axonal degeneration and improves NMJ function (Ackermann et al., 2013) as well as ameliorates survival and neuromuscular phenotype (Kaifer et al., 2017), possibly through the modulation of endocytic pathways (Hosseinibarkooie et al., 2016). However, it must be noted that a number of animal and patient studies do not reflect the suggested modifying powers of plastin 3 on SMA pathogenesis (McGovern et al., 2015; Stratigopoulos et al., 2010), highlighting the complex relationship that may exist between SMN and plastin 3. The pathological relevance and therapeutic importance of non-SMN targets can be highly dependent on the severity of the disease (Kaifer et al., 2017) and as such, should be evaluated in hypomorphic models, whether transgenically or pharmacologically induced. Nonetheless, the studies on RhoA-ROCK, PTEN and plastin 3 have highlighted actin modulators as potential targets for combinatorial therapeutic approaches for SMA.

Subsequent work has also revealed other promising non-SMN targets, including chondrolectin. It has been shown that this transmembrane protein (encoded by *Chodl*) and its binding partners are potential modifiers of axonal integrity in SMA mice and that altered expression of Chodl is found in spinal motor neurons of SMA mice (Bäumer et al., 2009). Importantly, increasing the expression of *chodl* rescues motor neuron outgrowth defects in a zebrafish model of SMA (Sleigh et al., 2014). Experiments in mouse models are underway to fully evaluate the therapeutic potential of Chodl modulation.

As described above, one of the housekeeping functions of SMN is the regulation of RNA metabolism, particularly in the biogenesis of snRNPs (Li et al., 2014), essential components of the RNA splicing machinery (Will and Lührmann, 2001). It has been demonstrated that SMN depletion specifically impacts the activity of the spliceosome complex containing the U12 snRNP (Doktor et al., 2017; Gabanella et al., 2007) and that the *Drosophila* *stasimon* (*stas*) gene is a direct target of U12-dependent splicing (Lotti et al., 2012). Stasimon (also known as Tmem41b in mammals) plays a role in synaptic transmission in neuronal synapses and its expression is significantly reduced and splicing similarly altered in motor and sensory neurons of SMA mice (Lotti et al., 2012). Importantly, overexpression of *stas* restored neurotransmitter release in *Drosophila smn* mutants and rescued motor axon growth and branching defects in an SMA zebrafish model (Lotti et al., 2012).

The activity of cyclin-dependent kinase 5 (Cdk5) has been reported to be upregulated in SMA mice and patient iPSC-derived motor neurons (Miller et al., 2015). The increased abundance of Cdk5 is responsible for the pathological hyperphosphorylation of the tau protein in SMN-depleted neuronal cells (Miller et al., 2015). A transgenic approach was used to completely knockout Cdk5 expression in SMA mice, which led to significant rescue of motor neuron synaptic stripping, motor neuron death and NMJ denervation (Miller et al., 2015). Interestingly, a recent unbiased RNA-sequencing-based assessment of global gene changes in SMN-depleted mouse tissues confirmed the specific missplicing of U12 snRNP-dependent genes, several of which are Ca^2+^ channel genes and may be upstream regulators of Cdk5 activity (Doktor et al., 2017), thus further highlighting Cdk5 as a potential pathological effector in SMA.

Finally, the ubiquitin-like modifier activating enzyme Uba1 (Groen and Gillingwater, 2015) and its downstream effectors [including the Wnt signaling effector β-catenin (Ctnnb1)], have been identified as major targets acting downstream of SMN to regulate neuromuscular and systemic pathology in SMA. Reduced levels of Uba1 were reported in all tissues and organs investigated from SMA mouse models (Aghamaleky Sarvestany et al., 2014; Wishart et al., 2014). Furthermore, β-catenin, which accumulates in neuromuscular tissues in SMA, has been uncovered as a key downstream target of Uba1 deficiencies in SMA. Importantly, pharmacological inhibition of β-catenin dramatically ameliorated neuromuscular pathology in zebrafish, *Drosophila* and mouse models of SMA (Wishart et al., 2014) while systemic *Uba1* gene therapy increased survival and improved neuromuscular and peripheral pathology of SMA mice (Powis et al., 2016).

Thus, a range of pathways are already candidates for non-SMN therapy approaches. As the list of molecular effectors grows, drug-screening approaches to identify pharmacological compounds that can modulate them will be essential. In addition, identifying novel targets should combine proteomics and transcriptomics studies with genome-based network analysis and drug-repositioning strategies. Combinatorial experimental paradigms should then be put in place to evaluate the therapeutic potential of a ‘cocktail treatment’ comprising SMN gene therapy and a non-SMN-targeting drug, optimally making use of the multiple *in vitro* and *in vivo* models discussed above (Fig. 3). While several non-SMN pathways and molecular targets have been highlighted as being aberrantly regulated in SMA models and display therapeutic potential, these studies remain, for the most part, in the pre-clinical discovery phase, in contrast to SMN-dependent strategies that are quickly dominating the clinical trial landscape. For non-SMN treatments to become a practical reality in the combinatorial approach paradigm, an efficient strategic plan needs to be established to facilitate their transition to the clinic.

## Improving systemic delivery of drugs to target CNS and non-CNS tissues

While motor neurons are undoubtedly the primary cellular target in SMA (Powis and Gillingwater, 2016), cumulative evidence highlights the role of other cells and tissues that may be clinically or sub-clinically affected. However, most of these studies have investigated these tissues or cells independently of the others. The hierarchal contribution of each to Type 0-IV SMA therefore remains unclear. While current gene therapies in clinical trials are promising, nusinersen (Ionis Pharmaceuticals/Biogen) delivery circumvents the peripheral tissues and organs by being injected directly to the CNS, and, although AAV9-*SMN1* gene therapy (AveXis) can be delivered systemically, multiple rounds of administration might not be possible due to immunogenicity (Basner-Tschakarjan and Mingozzi, 2014). In both of these cases, this raises the risk of incomplete rescue of SMN deficiency in peripheral organs and the potential for development of non-CNS pathologies later in life.

Development of novel therapeutic approaches targeting non-SMN targets should therefore include careful consideration of both CNS and systemic delivery methods. The optimal dosing regimen for a pharmacological compound should balance its ability to target all relevant tissues with the need to make therapy as non-invasive as possible. An option for systemic delivery of molecules is to conjugate them with a vehicle that can transport them across the membrane of multiple cell types. This has proven efficient for the delivery of ASOs under the neutrally charged chemistry of phosphorodiamidate morpholino (PMO) (Douglas and Wood, 2013). Cell-penetrating peptides (CPPs) have previously been shown to cross both plasma and endosomal membranes (Mitchell et al., 2000). One such peptide-conjugated PMO has been developed, termed peptide nucleic acids/PMO internalization peptide 6a (Pip6a)-PMO, that efficiently modulates splicing in various tissues of a DMD mouse model (Betts et al., 2012). Importantly, it is delivered via a single intravenous (IV) injection. Recently, it has been reported that conjugation of Pip6a to the *SMN2* ISS-N1 PMO results in dramatic improvements in survival and neuromuscular phenotype associated with increased FL-SMN levels in both CNS and peripheral tissues (Hammond et al., 2016). CPPs therefore have significant potential to facilitate targeting of SMN to the whole body as well the eventual delivery of therapeutic non-SMN targets or drugs in a similar fashion.

## Concluding remarks

**Fig. 4.**
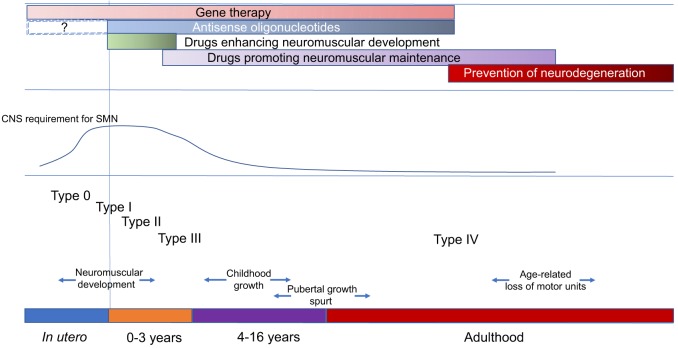
**Overview of the natural history of spinal muscular atrophy (SMA), major developmental milestones and treatment strategies.** Although the precise details of SMN expression in the developing human nervous system are difficult to study, evidence from animal models suggests that SMN levels peak in the period of maximum neuromuscular development and then decline to a stable low level. This means that there are different windows of opportunity for the various types of proposed therapies to be effectively employed. Whether combinatorial therapies might be particularly applicable in the more chronic phase of SMA or from the outset is a priority area for research. ASO therapy *in utero* will be dependent on an optimal route of delivery, which at present remains unclear.

Gene therapy and ASO approaches to increase SMN levels are now entering the clinical arena. In the severest form (Type I) of SMA, promising preliminary results must be balanced with a full appreciation of the potential limitations of such strategies. The value of SMN-based therapies in older Type II and III patients is unclear and it may be some time before these can be accurately evaluated. Translational research should therefore address the development of non-CNS and SMN-independent therapeutic approaches to complement and enhance the benefits of CNS-directed and SMN-dependent therapies, taking into account the need to maintain the neuromuscular system of an SMA patient through childhood and puberty, when there is maximal growth of the axial skeleton, and into adult life when the process of age-related attrition of motor units is likely to contribute to progressive loss of motor function (Fig. 4).

There remains a need for the use of various *in vitro* and *in vivo* models as well as molecular high-throughput approaches for the rapid identification of new targets and drugs. It will be crucial to develop tools to evaluate the effects of combination pharmacological therapies at different disease stages. It is therefore of utmost importance that the SMA research and clinical community, as well as those living with SMA, recognise the need to develop and test combinatorial therapeutic approaches that can be effectively delivered systemically and target both SMN and non-SMN molecular effectors. This will allow for a better understanding of the tissue requirements for SMN and non-SMN treatments and, ultimately, provide the best therapeutic strategy for SMA. As with most chronic progressive neurodegenerative disorders, it is likely that, once loss of neuronal integrity has been initiated, combinatorial approaches to therapy will be required to maintain neuromuscular health throughout life.
